# Supplementary material for: Reproductive Tract Microbial Transitions from Late Gestation to Early Postpartum Using 16S rRNA Metagenetic Profiling in First-Pregnancy Heifers
Source: Int J Mol Sci. 2024 Aug 23;25(17):9164. doi: 10.3390/ijms25179164 (PMC11394886; doi:10.3390/ijms25179164)
Supplement: Supplementary file 1 [file ijms-25-09164-s001.zip › ijms-3073401-supplementary.pdf]

Supplementary material

Reproductive Tract Microbial Transitions from Late Gestation to Early Postpartum Using 16S rRNA Metagenetic Profiling in First-Pregnancy Heifers

Shaked Druker [1,2], Ron Sicsic [1], Shachar Ravid [1], Shani Scheinin [1,2] and Tal Raz [1,3,*]

[1] Koret School of Veterinary Medicine, Robert H. Smith Faculty of Agriculture, Food and Environment, The Hebrew University of Jerusalem, Rehovot, Israel

[2] Hachaklait, Mutual Society for Veterinary Services, Caesarea Industrial Park, Caesarea, Israel

[3] Advanced Academic Programs, Krieger School of Arts and Sciences, Johns Hopkins University, Baltimore, MD, USA.

* Correspondence: tal.raz@mail.huji.ac.il

Table S1. Discriminative genera obtained from LEfSe comparison between Vag-pre and Vag-post samples. The logMaxMean represents the logarithm value of the maximum mean abundance of the OTU across the specified group (indicated as a class). LDA, Linear Discriminant Analysis score. Conditional formatting was applied (i.e., blue and green horizontal column bars) to logMaxMean and LDA values to visually represent the data.

Genus	logMaxMean	Class	LDA	P value
Trinickia	5.28	Vag-pre	4.86	<0.0001
Micrococcaceae_unclassified	4.87	Vag-pre	4.18	0.0071
Intrasporangiaceae_unclassified	4.42	Vag-pre	3.75	0.0019
Staphylococcus	4.00	Vag-pre	3.65	0.0009
Betaproteobacteria_unclassified	4.06	Vag-pre	3.58	<0.0001
Paraburkholderia	3.89	Vag-pre	3.48	0.0002
Burkholderiales_unclassified	3.85	Vag-pre	3.44	0.0001
Kocuria	4.02	Vag-pre	3.40	0.0040
Micrococcales_unclassified	4.14	Vag-pre	3.38	0.0030
Uruburuella	3.65	Vag-pre	3.28	0.0006
Burkholderiaceae_unclassified	3.62	Vag-pre	3.24	0.0001
Lactobacillales_unclassified	3.73	Vag-pre	3.23	0.0001
Chitinophagaceae_unclassified	3.86	Vag-pre	3.20	0.0347
Pseudomonadaceae_unclassified	3.63	Vag-pre	3.16	0.0015
Proteobacteria_unclassified	3.64	Vag-pre	3.12	0.0003
Enterobacteriaceae_unclassified	3.43	Vag-pre	3.06	0.0002
Rhodanobacter	3.49	Vag-pre	3.03	0.0005
Corynebacterium	3.56	Vag-pre	3.02	0.0012
Rhizobiales_unclassified	3.73	Vag-pre	3.00	0.0432
Vibrionaceae_unclassified	2.53	Vag-pre	2.94	0.0168
Tersicoccus	3.56	Vag-pre	2.92	0.0079
Oceanotoga	3.27	Vag-pre	2.91	0.0039
Enterococcus	3.18	Vag-pre	2.89	0.0035
Mycobacteriales_unclassified	3.24	Vag-pre	2.89	0.0002
Gammaproteobacteria_unclassified	3.53	Vag-pre	2.87	0.0072
Micrococcus	3.28	Vag-pre	2.87	0.0403
Boudabousia	2.87	Vag-pre	2.87	0.0165
Burkholderia	2.95	Vag-pre	2.83	0.0001
Staphylococcaceae_unclassified	3.27	Vag-pre	2.82	0.0110
Bradyrhizobiaceae_unclassified	3.49	Vag-pre	2.81	0.0283
Undibacterium	2.67	Vag-pre	2.80	0.0004
Rhodanobacteraceae_unclassified	3.18	Vag-pre	2.79	0.0009
Enterobacterales_unclassified	2.71	Vag-pre	2.79	0.0051
Paracoccus	2.61	Vag-pre	2.78	0.0082
Neisseriaceae_unclassified	3.31	Vag-pre	2.78	0.0156
Enterococcaceae_unclassified	3.07	Vag-pre	2.77	0.0090
Sphingomonadaceae_unclassified	3.23	Vag-pre	2.77	0.0047
Bacilli_unclassified	3.18	Vag-pre	2.77	0.0089
Salinivibrio	2.50	Vag-pre	2.75	0.0047
Carnobacteriaceae_unclassified	2.57	Vag-pre	2.71	0.0015
Lawsonella	2.79	Vag-pre	2.71	0.0034

Halomonas	2.57	Vag-pre	2.69	0.0480
Pseudomonas	2.93	Vag-pre	2.66	0.0150
Chryseobacterium	2.50	Vag-pre	2.63	0.0245
Devosiaceae_unclassified	2.95	Vag-pre	2.60	0.0314
Actinobacteria_unclassified	3.03	Vag-pre	2.56	0.0307
Cyanobacteria	2.74	Vag-pre	2.55	0.0292
Xanthomonadales_unclassified	2.91	Vag-pre	2.55	0.0020
Oxalobacteraceae_unclassified	2.65	Vag-pre	2.52	0.0354
Rhizomicrobium	3.36	Vag-pre	2.49	0.0372
Lactococcus	2.72	Vag-pre	2.43	0.0343
Sphingobacteriaceae_unclassified	2.51	Vag-pre	2.43	0.0488
Phyllobacteriaceae_unclassified	2.62	Vag-pre	2.36	0.0293
Fusobacterium	5.10	Vag-post	4.53	0.0003
Leptotrichiaceae_unclassified	4.67	Vag-post	4.38	0.0123
Histophilus	4.56	Vag-post	4.23	0.0223
Mycoplasmopsis	4.78	Vag-post	4.20	0.0425
Parvimonas	4.29	Vag-post	3.93	0.0056
Bacteroidales_unclassified	4.33	Vag-post	3.77	0.0083
Fusobacteriaceae_unclassified	4.43	Vag-post	3.65	0.0179
Caviibacter	3.95	Vag-post	3.52	<0.0001
Acinetobacter	3.89	Vag-post	3.22	0.0120
Peptostreptococcus	3.64	Vag-post	3.15	0.0149
Fusobacteriales_unclassified	3.56	Vag-post	3.05	0.0001
Mollicutes_unclassified	2.95	Vag-post	2.68	0.0017

Table S2. Discriminative genera obtained from LEfSe comparison between Vag-pre and Utr-post samples. The logMaxMean represents the logarithm value of the maximum mean abundance of the OTU across the specified group (indicated as a class). LDA, Linear Discriminant Analysis score. Conditional formatting was applied (i.e., blue and green horizontal column bars) to logMaxMean and LDA values to visually represent the data. *uncl.*, unclacified.

Genus	logMaxMean	Class	LDA	P value
Trinickia	5.28	Vag-pre	4.93	<0.0001
Micrococcaceae_unclassified	4.87	Vag-pre	4.57	<0.0001
Intrasporangiaceae_unclassified	4.42	Vag-pre	4.10	<0.0001
Ruminococcaceae_unclassified	4.47	Vag-pre	3.92	0.0003
Glutamicibacter	4.26	Vag-pre	3.91	<0.0001
Micrococcales_unclassified	4.14	Vag-pre	3.84	<0.0001
Streptococcus	4.30	Vag-pre	3.76	0.0003
Kocuria	4.02	Vag-pre	3.76	<0.0001
Oerskovia	4.02	Vag-pre	3.75	<0.0001
Lachnospiraceae_unclassified	4.37	Vag-pre	3.68	0.0011
Betaproteobacteria_unclassified	4.06	Vag-pre	3.65	<0.0001
Paraburkholderia	3.89	Vag-pre	3.56	<0.0001
Staphylococcus	4.00	Vag-pre	3.56	<0.0001
Burkholderiales_unclassified	3.85	Vag-pre	3.50	<0.0001
Acinetobacter	3.82	Vag-pre	3.49	<0.0001

Uruburuella	3.65	Vag-pre	3.33	<0.0001
Lactobacillales_unclassified	3.73	Vag-pre	3.33	<0.0001
Burkholderiaceae_unclassified	3.62	Vag-pre	3.30	<0.0001
Chitinophagaceae_unclassified	3.86	Vag-pre	3.29	<0.0001
Tersicoccus	3.56	Vag-pre	3.28	<0.0001
Pseudomonadaceae_unclassified	3.63	Vag-pre	3.28	<0.0001
Corynebacterium	3.56	Vag-pre	3.23	<0.0001
Actinobacteria_unclassified	3.81	Vag-pre	3.22	0.0001
Rhizobiales_unclassified	3.73	Vag-pre	3.14	<0.0001
Rhodanobacter	3.49	Vag-pre	3.11	<0.0001
Alphaproteobacteria_unclassified	3.65	Vag-pre	3.09	0.0001
Proteobacteria_unclassified	3.64	Vag-pre	3.08	<0.0001
Cellulomonadaceae_unclassified	3.33	Vag-pre	3.08	<0.0001
Phocaeicola	3.69	Vag-pre	3.07	0.0068
Dietzia	3.30	Vag-pre	3.05	<0.0001
Neisseriaceae_unclassified	3.31	Vag-pre	3.05	0.0003
Devosia	3.45	Vag-pre	2.99	0.0001
Micrococcus	3.28	Vag-pre	2.98	<0.0001
Rhodobacteraceae_unclassified	3.39	Vag-pre	2.97	<0.0001
Gammaproteobacteria_unclassified	3.53	Vag-pre	2.96	0.0004
Bradyrhizobiaceae_unclassified	3.49	Vag-pre	2.96	0.0004
Mycobacteriales_unclassified	3.24	Vag-pre	2.94	<0.0001
Oceanotoga	3.27	Vag-pre	2.93	<0.0001
Enterococcus	3.18	Vag-pre	2.92	<0.0001
Staphylococcaceae_unclassified	3.27	Vag-pre	2.91	<0.0001
Enterobacteriaceae_unclassified	3.43	Vag-pre	2.85	<0.0001
Turicibacter	3.28	Vag-pre	2.85	0.0002
Enterococcaceae_unclassified	3.07	Vag-pre	2.84	<0.0001
Romboutsia	3.30	Vag-pre	2.83	0.0001
Burkholderia	2.95	Vag-pre	2.83	<0.0001
Rhizomicrobium	3.36	Vag-pre	2.82	0.0006
Clostridiales_Incertae_Sedis_XIII_unclas	3.30	Vag-pre	2.82	0.0001
Moraxellaceae_unclassified	2.90	Vag-pre	2.80	<0.0001
Pseudomonas	2.93	Vag-pre	2.80	<0.0001
Paeniclostridium	3.22	Vag-pre	2.79	0.0007
Sphingomonadaceae_unclassified	3.23	Vag-pre	2.78	<0.0001
Psychrobacter	2.73	Vag-pre	2.77	0.0002
Salinivibrio	2.50	Vag-pre	2.76	0.0001
Boudabousia	2.87	Vag-pre	2.76	<0.0001
Acetoanaerobium	2.82	Vag-pre	2.75	<0.0001
Asticcacaulis	3.13	Vag-pre	2.75	0.0126
Nocardioidaceae_unclassified	2.33	Vag-pre	2.74	0.0082
Paracoccus	2.61	Vag-pre	2.74	0.0002
Opitutaceae_unclassified	3.22	Vag-pre	2.74	0.0003
Undibacterium	2.67	Vag-pre	2.73	<0.0001
Halomonadaceae_unclassified	2.39	Vag-pre	2.73	0.0002

Clostridiaceae_1_unclassified	3.16	Vag-pre	2.72	0.0081
Rhodanobacteraceae_unclassified	3.18	Vag-pre	2.72	<0.0001
Paenibacillaceae_1_unclassified	3.09	Vag-pre	2.71	0.0001
Halomonas	2.57	Vag-pre	2.70	0.0002
Aerococcus	2.86	Vag-pre	2.70	0.0005
Brevundimonas	2.90	Vag-pre	2.69	0.0022
Brachybacterium	2.83	Vag-pre	2.67	<0.0001
Oxalobacteraceae_unclassified	2.65	Vag-pre	2.66	<0.0001
Lactococcus	2.72	Vag-pre	2.64	<0.0001
Xanthomonadales_unclassified	2.91	Vag-pre	2.64	0.0012
Ornithinimicrobium	2.55	Vag-pre	2.64	0.0002
Enterobacterales_unclassified	2.71	Vag-pre	2.63	0.0001
Planococcaceae_unclassified	2.57	Vag-pre	2.63	0.0029
Vibrionaceae_unclassified	2.53	Vag-pre	2.62	0.0003
Clostridium_sensu_stricto	3.29	Vag-pre	2.62	0.0091
Gp6_unclassified	2.85	Vag-pre	2.62	0.0008
Lawsonella	2.79	Vag-pre	2.61	<0.0001
Bacilli_unclassified	3.18	Vag-pre	2.61	0.0414
Chryseobacterium	2.50	Vag-pre	2.60	0.0010
Enhydrobacter	2.62	Vag-pre	2.60	0.0123
Actinobacteria_unclassified	3.03	Vag-pre	2.60	<0.0001
Opitutus	3.07	Vag-pre	2.60	0.0007
Dokdonella	3.03	Vag-pre	2.60	0.0133
Carnobacteriaceae_unclassified	2.57	Vag-pre	2.60	<0.0001
Mogibacterium	2.67	Vag-pre	2.57	0.0004
Cytophagales_unclassified	2.97	Vag-pre	2.57	0.0035
Comamonadaceae_unclassified	3.05	Vag-pre	2.56	0.0006
Deltaproteobacteria_unclassified	2.85	Vag-pre	2.55	0.0225
Devosiaceae_unclassified	2.95	Vag-pre	2.54	0.0053
Sphingopyxis	2.92	Vag-pre	2.54	0.0011
Cyanobacteria /Chloroplast _ unclassified	2.74	Vag-pre	2.54	0.0019
Rhizobiaceae_unclassified	2.92	Vag-pre	2.53	0.0048
Olsenella	2.87	Vag-pre	2.53	0.0025
Erythrobacteraceae_unclassified	2.64	Vag-pre	2.51	0.0080
Brevibacterium	2.63	Vag-pre	2.51	0.0009
Micropepsaceae_unclassified	3.10	Vag-pre	2.51	0.0187
Anaerobutyricum	2.60	Vag-pre	2.50	0.0037
Polyangiaceae_unclassified	2.95	Vag-pre	2.49	0.0182
Streptomycetaceae_unclassified	2.90	Vag-pre	2.47	0.0153
Rhodospirillales_unclassified	3.04	Vag-pre	2.46	0.0016
Sphingomonadales_unclassified	2.62	Vag-pre	2.46	0.0144
Streptacidiphilus	2.77	Vag-pre	2.43	0.0334
Flavobacteriaceae_unclassified	2.62	Vag-pre	2.43	0.0083
Phyllobacteriaceae_unclassified	2.62	Vag-pre	2.39	0.0076
Microbacteriaceae_unclassified	2.52	Vag-pre	2.34	0.0412

Sphingobacteriaceae_unclassified	2.51	Vag-pre	2.30	0.0039
Gemella	4.94	Utr-post	4.59	0.0241
Leptotrichiaceae_unclassified	4.56	Utr-post	4.18	0.0095
Trueperella	4.12	Utr-post	3.90	0.0063
Streptobacillus	4.16	Utr-post	3.73	0.0039
Caviibacter	3.26	Utr-post	2.94	0.0009
Gemmatimonadaceae_unclassified	3.11	Utr-post	2.76	0.0336

Table S3: Discriminative genera obtained from LEfSe comparison between Vag-post and Utr-post samples. The logMaxMean represents the logarithm value of the maximum mean abundance of the OTU across the specified group (indicated as a class). LDA, Linear Discriminant Analysis score. Conditional formatting was applied (i.e., blue and green horizontal column bars) to logMaxMean and LDA values to visually represent the data. *uncl.*, unclacified.

Genus	logMaxMean	Class	LDA	P value
Trinickia	4.79	Vag-post	4.41	<0.0001
Leptotrichiaceae_unclassified	4.67	Vag-post	4.24	0.0021
Micrococcaceae_unclassified	4.38	Vag-post	4.11	<0.0001
Ruminococcaceae_unclassified	4.47	Vag-post	3.90	0.0032
Fusobacteriaceae_unclassified	4.43	Vag-post	3.81	0.0057
Clostridiales_unclassified	4.50	Vag-post	3.81	0.0312
Lachnospiraceae_unclassified	4.40	Vag-post	3.78	0.0012
Intrasporangiaceae_unclassified	4.00	Vag-post	3.67	<0.0001
Glutamicibacter	3.97	Vag-post	3.65	0.0001
Acinetobacter	3.89	Vag-post	3.62	0.0016
Oerskovia	3.80	Vag-post	3.51	<0.0001
Micrococcales_unclassified	3.78	Vag-post	3.46	<0.0001
Chitinophagaceae_unclassified	3.62	Vag-post	3.26	0.0026
Kocuria	3.47	Vag-post	3.21	0.0002
Phocaeicola	3.63	Vag-post	3.19	0.0197
Peptostreptococcus	3.64	Vag-post	3.16	0.0467
Paraburkholderia	3.46	Vag-post	3.12	0.0001
Alphaproteobacteria_unclassified	3.49	Vag-post	3.04	0.0062
Burkholderiales_unclassified	3.41	Vag-post	3.03	0.0001
Betaproteobacteria_unclassified	3.67	Vag-post	3.02	0.0031
Actinobacteria_unclassified	3.70	Vag-post	2.98	0.0113
Rhizobiales_unclassified	3.55	Vag-post	2.94	0.0085
Uruburuella	3.25	Vag-post	2.88	0.0001
Corynebacterium	3.20	Vag-post	2.88	0.0002
Cellulomonadaceae_unclassified	3.12	Vag-post	2.87	<0.0001
Micrococcus	3.06	Vag-post	2.86	0.0001
Tersicoccus	2.98	Vag-post	2.80	0.0004
Lactobacillales_unclassified	3.28	Vag-post	2.79	0.0004
Dietzia	3.02	Vag-post	2.77	0.0013
Burkholderiaceae_unclassified	3.17	Vag-post	2.75	0.0020
Paeniclostridium	3.27	Vag-post	2.75	0.0010
Rhodanobacter	3.17	Vag-post	2.72	0.0007

Clostridium_sensu_stricto	3.33	Vag-post	2.72	0.0366
Paenibacillaceae_1_unclassified	2.95	Vag-post	2.72	0.0006
Pseudomonadaceae_unclassified	3.18	Vag-post	2.72	0.0002
Moraxellaceae_unclassified	2.75	Vag-post	2.69	0.0023
Burkholderia	2.24	Vag-post	2.69	0.0032
Devosia	3.18	Vag-post	2.68	0.0014
Staphylococcaceae_unclassified	2.73	Vag-post	2.67	0.0115
Oceanotoga	2.85	Vag-post	2.66	0.0009
Ihubacter	3.19	Vag-post	2.66	0.0497
Mollicutes_unclassified	2.95	Vag-post	2.62	0.0077
Staphylococcus	3.12	Vag-post	2.62	0.0084
Enterococcaceae_unclassified	2.81	Vag-post	2.62	0.0018
Enterococcus	2.65	Vag-post	2.60	0.0006
Pseudomonas	2.38	Vag-post	2.60	0.0005
Clostridiales_Incertae_Sedis_XIII_unclas	3.13	Vag-post	2.59	0.0189
Opitutaceae_unclassified	3.17	Vag-post	2.59	0.0072
Turicibacter	3.05	Vag-post	2.57	0.0008
Gp6_unclassified	2.77	Vag-post	2.56	0.0132
Brachybacterium	2.50	Vag-post	2.56	0.0005
Romboutsia	3.01	Vag-post	2.55	0.0038
Harryflintia	2.85	Vag-post	2.50	0.0489
Peptostreptococcaceae_unclassified	2.82	Vag-post	2.48	0.0112
Rhizobiaceae_unclassified	2.78	Vag-post	2.48	0.0281
Mycobacteriales_unclassified	2.78	Vag-post	2.46	0.0008
Streptomycetaceae_unclassified	2.71	Vag-post	2.46	0.0079
Anaerotignum	2.89	Vag-post	2.44	0.0179
Sphingopyxis	2.80	Vag-post	2.44	0.0298
Mogibacterium	2.40	Vag-post	2.43	0.0066
Opitutus	2.79	Vag-post	2.42	0.0299
Comamonadaceae_unclassified	2.88	Vag-post	2.38	0.0221
Anaerobutyricum	2.37	Vag-post	2.34	0.0214
Actinobacteria_unclassified	2.75	Vag-post	2.32	0.0060
Myxococcales_unclassified	2.46	Vag-post	2.21	0.0245
Trueperella	4.12	Utr-post	3.79	0.0442
Streptococcus	4.15	Utr-post	3.70	0.0080
Peptoniphilus	4.04	Utr-post	3.55	0.0378
Rhodobacteraceae_unclassified	3.18	Utr-post	2.85	0.0035
Gemmatimonadaceae_unclassified	3.11	Utr-post	2.68	0.0103
Rhodospirillales_unclassified	2.86	Utr-post	2.61	0.0088
